# Human Epidermal Growth Factor Receptor 2 [HER‐2/neu] Amplification and Microsatellite Stable Status in Gastric and Gastroesophageal Adenocarcinoma: Assessing Frequency and Prognostic Implications at the Cancer Institute of Iran

**DOI:** 10.1002/cnr2.70314

**Published:** 2025-08-25

**Authors:** Samaneh Salarvand, Abbas Mohammadi, Reza Shahsiah, Farzaneh Bagheri, Mahsa Gholizadeh, Somayeh Jolany‐Vangah, Ebrahim Esmati, Marzieh Lashkari, Vahid Soleymani, Seraj Zareh‐Dehabadi, Vahid Mehrtash, Amirmohsen Jalaeefar, Mohammad Shirkhoda, Moones Toosang, Romina Abyaneh, Reza Ghalehtaki

**Affiliations:** ^1^ Department of Clinical and Anatomical Pathology, Cancer Institute, School of Medicine Tehran University of Medical Sciences Tehran Iran; ^2^ Department of Ophthalmology, School of Medicine Ahvaz Joundishapur University of Medical Sciences Ahvaz Iran; ^3^ Department of Radiation Oncology, Cancer Institute, IKHC, School of Medicine Tehran University of Medical Sciences Tehran Iran; ^4^ Radiation Oncology Research Center, Cancer Research Institute Tehran University of Medical Sciences Tehran Iran; ^5^ Department of Oncosurgery, Cancer Institute, IKHC Tehran University of Medical Sciences Tehran Iran

**Keywords:** Adenocarcinoma, DNA mismatch repair, Gastric neoplasm, HER family receptor

## Abstract

**Background:**

Molecular targeted therapy and immunotherapy have shown promise in managing gastric adenocarcinoma. The amplified expression of Human epidermal growth factor receptor‐2 (HER‐2) and microsatellite stable (MSI) status serve as indicators of response to targeted therapy and immunotherapy, respectively.

**Aims:**

This study assessed the frequency of MSI status and HER‐2 expression in a pretreated sample of Iranian patients with gastric and gastroesophageal (GE) adenocarcinoma.

**Methods and Results:**

We conducted HER‐2/neu expression and mismatch repair (MMR) system analyses on specimens from patients with gastric and GE adenocarcinoma at the Cancer Institute of Iran. Archival tissues from 135 mainly pre‐treated surgical specimens of gastric adenocarcinoma cases were used for HER‐2 analysis, and 66 specimens were used for MSI analysis. All specimens were tested for HER‐2 amplification, revealing that 11 patients (8.1%) had HER‐2 amplification, and three out of 66 examined patients exhibited MSI‐H. Patients with HER‐2 overexpressed specimens had a shorter median overall survival (OS) compared with HER‐2 negative cases (21 months (95% CI: 1.4–40.6) vs. 31 months (95% CI: 22.9–39), *p* = 0.18). The median disease‐free survival (DFS) for HER‐2 positive and negative patients was 15 and 28 months, respectively (*p* = 0.25). The estimated median OS and DFS for patients with negative MSI were 26 and 20 months, respectively. However, none of the patients with MSI‐positive status experienced recurrence, metastases, or death within the follow‐up period; thus, MSI‐H patients had a significantly improved OS and DFS (*p* = 0.018 and 0.020).

**Conclusion:**

HER‐2 expression, while less common in our Iranian population compared with North American or Western European countries, showed a nonsignificant trend toward poor outcomes in patients with gastric adenocarcinoma. MSI‐H status was highly infrequent in our population, suggesting that immunotherapy may not be a beneficial treatment for a significant fraction of Iranian patients with gastric adenocarcinoma. However, a minority may still benefit from it. MSI‐H was associated with reduced perineural invasion and improved OS and DFS. Therefore, this hypothesis warrants further investigation in clinical trials to underscore the prognostic significance of HER‐2 and MSI status and the value of molecular profiling in guiding personalized treatment strategies.

## 1 Introduction

Gastric cancer was responsible for 1.1 million new cases and 800 000 deaths in 2020, ranking it as the fifth most prevalent cancer and the fourth cause of death due to cancer worldwide, except for non‐melanoma skin cancer. The high‐incidence regions were Eastern Asia and Eastern Europe, while Northern America and Northern Europe had similar rates to those in Africa, which were generally low [1, 2]. The occurrence and death rates of non‐cardia gastric cancer have decreased over the last 50 years, while the relative increase in gastric cardia cancers has stabilized, at least in the Netherlands and the United States [1, 2, 3, 4, 5, 6]. A fresh and opposing discovery has surfaced, which necessitates additional verification. The incidence of gastric cancer, both cardia and non‐cardia, has risen in young adults who are 50 years old or younger in both high‐ and low‐risk countries, especially males [7, 8].

Esophageal and gastric cancers need a multi‐modality treatment approach, including surgery, chemotherapy, and radiotherapy [9, 10]. However, with current treatments, the survival rate of patients with advanced cancer remains extremely low [10]. To date, several next‐generation sequencing (NGS) studies have uncovered several genes that are commonly mutated in gastric cancer [11, 12], encouraging the development of targeted gene therapy that effectively facilitates the control of gastric cancer patients and improves overall survival (OS) [13, 14].

Human epidermal growth factor receptor‐2 (HER‐2), referred to as erb‐b2 receptor tyrosine kinase 2 (ERBB2) as well, is a receptor for human growth factor that governs the differentiation and growth of cells [15]. Elevated levels of HER‐2 amplification can stimulate the excessive expression of proteins on the cell membrane, consequently leading to the acquisition of malignant characteristics by the cells [16]. Trastuzumab is a pharmaceutical agent that specifically targets the HER‐2 protein, aiming to enhance the survival rate of patients diagnosed with localized and metastatic HER‐2 positive breast cancer [17]. HER‐2 mutations are commonly observed in diverse forms of cancer, including breast, lung, and gastric cancer [18]. The incidence of HER‐2 positivity in gastric cancer rises with age and positively correlates with the intestinal type [19]. The manifestation of HER‐2 protein is also linked to Lauren classification, tumor differentiation, Borrmann type, and P53 expression in gastric cancer [20]. Numerous studies have demonstrated that patients with HER‐2 positive tumors tend to have an unfavorable prognosis in comparison to patients with HER‐2 negative tumors [21, 22]. In contrast to breast cancer, the relationship between HER‐2 and prognosis in gastric cancer patients remains a subject of debate. Some investigations have suggested a strong association between HER‐2 positive tumors and a significantly worse prognosis, whereas others have found no correlation between HER‐2 status and prognosis [22, 23, 24, 25, 26].

Microsatellites are short, repeated sequences of DNA [27]. Microsatellite instability (MSI) reflects a deficient mismatch repair system [28]. MSI is recognized as a favorable prognostic biomarker that is particularly linked to a good prognosis in various cancers, notably colorectal cancer (CRC) and gastric cancer [29, 30, 31].

The prognostic implications of MSI and HER‐2 amplification differ significantly. While HER‐2 amplification is associated with an unfavorable prognosis, MSI is linked to a favorable prognosis [32, 33]. In individuals affected by HER‐2 positive gastric cancer, the inclusion of trastuzumab in first‐line chemotherapy has been observed to enhance the survival rate [17, 32, 33]. As a result, HER‐2 targeted therapy has been designated as the primary treatment approach for HER‐2 positive patients with gastric cancer. Furthermore, MSI is also taken into consideration in the context of adjuvant immunotherapy [32, 33]. However, the potential synergistic effects of these two indicators in gastric cancer remain largely unexplored.

Considering the concerns above, the presence of HER‐2 expression and MSI serves as an indicator of the response to these innovative therapies. Consequently, this investigation endeavored to evaluate the manifestation of MSI phenotype and HER‐2 expression in Iranian individuals with gastroesophageal (GE) and gastric adenocarcinoma. This pioneering study may provide a suitable approximation to inform us about the proportion of patients in the GE or gastric cancer population who may benefit from these agents.

## 2 Methods

### 2.1 Patients and Specimens

In this retrospective cohort study, we analyzed patients with gastric or GE adenocarcinoma referred to the Cancer Institute of Iran, affiliated with Tehran University of Medical Sciences, between 2018 and 2023. All pre‐treatment diagnoses were pathologically confirmed after the biopsy specimens were examined. Our inclusion and exclusion criteria led us to utilize all available archival formalin‐fixed paraffin‐embedded tissues from resected specimens of patients with gastric adenocarcinoma without gender or age restrictions.

Our study design acquired approval from the institutional review board (Grant no. 97‐03‐30‐40 246, 97‐01‐207‐38 089, and 95‐04‐207‐33 772) and was reviewed and approved by the local ethics committee (Ethics code: IR.TUMS.VCR.REC.1397.020, IR.TUMS.IKHC.REC.1397.325). All patients gave written informed consent to publish case details and use images. The disease stage, grade, survival rate, and treatment response were meticulously recorded in designated data forms. The analysis comprised tissue samples collected during surgery from patients suffering from esophageal and gastric cancers. For all cases, we thoroughly reviewed the location of the primary tumor, histological grade, the extent of infiltration, lymphatic and venous invasion, surgical margin, surgical stage, TRG, type of neoadjuvant therapy, HER‐2 status, and MSI phenotype.

## 3 Laboratory Techniques

### 3.1 HER‐2

**FIGURE 1:**
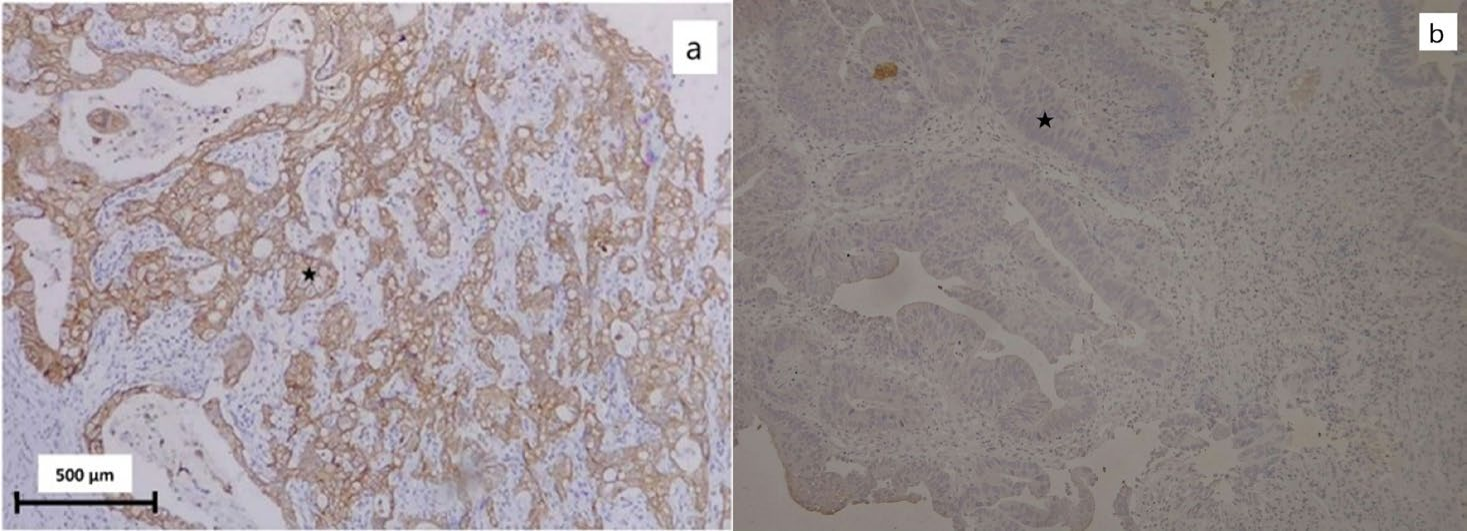
(a) Gastric adenocarcinoma with positive HER‐2 on IHC, Complete membrane intense staining in more than 10% of tumor cells (×100). (b) Gastric adenocarcinoma with negative HER‐2 on IHC, tumoral cells show no staining (×100), 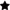 tumoral cells.

**FIGURE 2:**
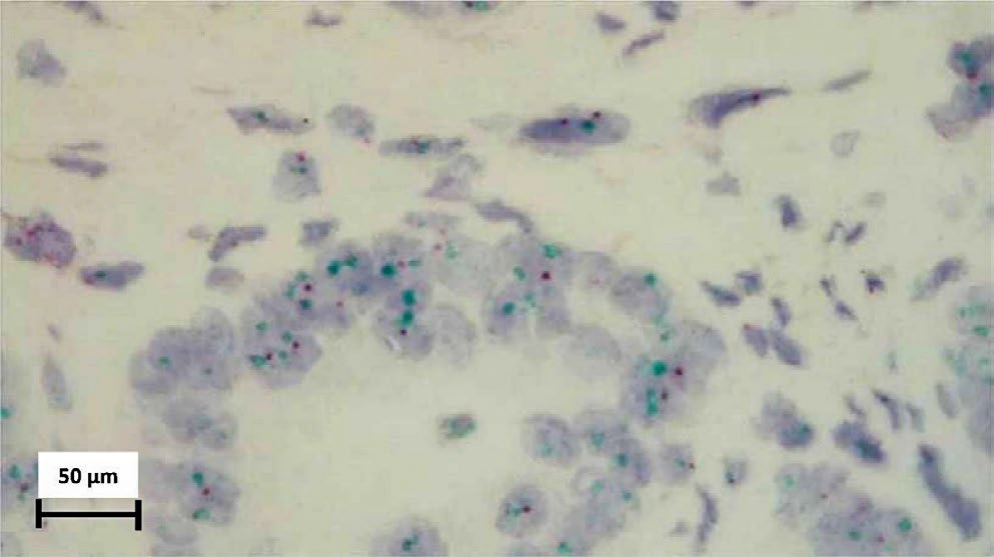
Figure represents a HER‐2 gene amplified specimen (HER‐2/CEN‐17 ratio > 2.0). Green dots: HER‐2 gene, Red dots: CEN‐17 (×400).

The standard and routine methods were followed during the tissue preparation process, which included initial tissue incision, fixation, processing with a tissue processor, microtome incisions, slide preparation, staining, and assembly. The marked tumor sites on the slides were matched with the paraffin block under a microscope. To detect HER‐2/neu amplification, immunohistochemistry (IHC) was used. The IHC reactions employed the use of the streptavidin‐biotin‐peroxidase complex technique (StreptABC, DAKO, Denmark). The tissue sections were transferred for antigen retrieval in a pressure cooker with citrate buffer after de‐paraffinization. Endogenous peroxidase activity was inhibited using a 3% H_2_O_2_ solvent. The sections were then exposed to polyclonal primary antibodies against HER‐2/neu (1:500, A0485, DAKO, Denmark), followed by incubation with a secondary biotinylated antibody from LSAB+ peroxidase Kit (DAKO, K0690, Denmark), and subsequent incubation with Streptavidin HRP (DAKO, Denmark). Hematoxylin was used for counterstaining (Figure 1). The intensity of tumor cells and the staining pattern were used to describe the immunohistochemical analyses of HER‐2 expression. The chromogenic in situ hybridization (CISH) method was also utilized for equivalent results (as shown in Figure 2).

We manually examined the HER‐2 amplification status using the HER‐2/CEN17 dual CISH detection kit (Zytovision technology) on paraffin‐embedded sections. Initially, positively charged slides with overlying paraffin sections were set in an oven at 60°C overnight. Subsequently, they underwent de‐waxing by floating in xylene and graded ethanols, followed by a final rinse in distilled water. For temperature pretreatment, the slides were exposed to an EDTA pH: 8 buffer solution for 20 min at 95°C; then washed with TBS buffer at room temperature and air‐dried. Enzymatic digestion was performed using 100 μL of ready‐to‐use pepsin solution for 7 min at 37°C. After a subsequent wash with TBS buffer and Tween 20 at room temperature, the slides were dehydrated in graded alcohols reversely and air‐dried.

Following this, 10 μL of HER‐2/CEN17 CISH probe was applied over the tissue, and a coverslip was placed on the section, sealed with rubber cement. The hybridization step involved denaturation on a hotplate and incubation in a humid chamber for 16 h at 37°C. Then, the coverslips were withdrawn, and the slides were washed and stored in TBS solution until the detection steps were performed. After applying a peroxidase‐blocking solution, a mix of secondary antibodies and polymers was applied. This was followed by incubation with red chromogen for 10 min and then with green chromogen. After each step, the specimens were washed three times using distilled water. In the final step, staining was performed with hematoxylin solution for 30 s, followed by bluing, dehydration, and clearing using concentrated alcohols and xylene, and then the samples were mounted. The results were then interpreted.

### 3.2 MSI

To assess the MSI status, it was necessary to conduct DNA isolation and PCR tests. The tissue DNA was extracted using the QIAGEN Read TM DNA FFPE Kit, and the extraction process was carried out following the kit instructions. The quality of DNA was evaluated based on the absorbance ratios at 260 nm/280 nm. In this study, a pentaplex PCR was employed to detect coding genes for MMR protein expression. We used suitable primers to amplify five human DNA microsatellite sequences based on the Bethesda panel of markers. The sequences for amplifying NR27, BAT26, BAT25, NR24, and NR21 were obtained from Macrogen (Geum Chun‐Gu, Seoul, Korea) with their previously defined sequences [34]. The PCR reaction was executed using a LightCycler Nano machine (Roche Diagnostics GmbH, Mannheim, Germany). The melting graphs were evaluated, and if acceptable, PCR products were then transferred to capillary electrophoresis according to the manufacturer's instructions. The electropherograms were analyzed using Peak Scanner software version 1.0 (Applied Biosystems, CA). Instability at more than one locus was determined as MSI‐H; instability at a single locus was defined as low MSI (MSI‐L), and no instability at any locus was defined as MSS for interpretation.

## 4 Statistical Analysis

All available surgical samples were subjected to laboratory tests within a predetermined time frame to identify specific mutations or expressions. This study did not employ power or sample size analysis. Group comparisons were conducted using the Student's *t*‐test and chi‐square test, as appropriate. OS time was calculated by measuring the time between surgery and the patient's last follow‐up or death. Disease‐free survival (DFS) was calculated by measuring the time between surgery and the patient's last follow‐up without any complications, or until recurrence or death. The Kaplan–Meier method was used to estimate median survival in months, and *p* values for comparisons among subgroups were calculated using the log‐rank test. Kaplan–Meier curves for OS and DFS in subgroups were generated using R software (version 4.4) with the survival, survminer, ggplot2, and dplyr packages. All other statistical analyses were performed using SPSS software (version 27).

## 5 Results

**FIGURE 3:**
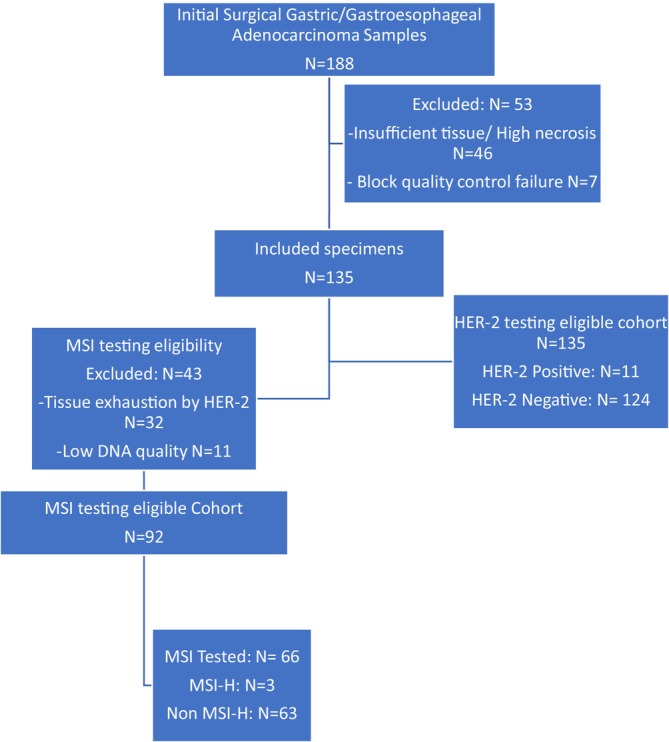
Flowchart of sample inclusion and exclusion.

A total of 188 initial surgical specimens of gastric or GE adenocarcinoma were collected. After quality assessment, 53 specimens were excluded due to insufficient tissue or high necrosis (*n* = 46) and block quality control failure (*n* = 7). This yielded 135 eligible specimens that were included in the study for further analysis (Figure 3). The final cohort had a mean age of 62.96 ± 9.66 years, with a median of 63.5 years (range: 36–84). Notably, 89% of the gastric cancer patients were over 50 years old. The male‐to‐female ratio was 2.29:1. The most common location of the tumor was the gastric cardia and gastroesophageal junction (GEJ), accounting for 53% of cases. The intestinal subtype accounted for the predominant histological type of adenocarcinoma, with 78.5% of cases. Poor histological differentiation was present in 40.7% of the tumors. The majority of patients had locally‐advanced‐stage disease.

The clinical stages were distributed as follows: IB (5.2%), IIA (5.9%), IIB (6.7%), IIIA (16.3%), IIIB (11.1%), IIIC (3%), and IV (1.5%). Clinical stage data were not available for 68 (50.3%) patients. Exactly 133 patients underwent curative surgery, and two patients underwent open‐closure surgery for peritoneal infiltration between neoadjuvant treatment and surgery. Table 1 presents the characteristics of the study population, including demographic information and clinical profiles.

**TABLE 1:** Baseline and pathological characteristics of patients.

Characteristic	Total, *N* (%)	HER‐2		
		Positive	Negative	*p*
Age				
Year (mean ± SD)	62.96 ± 9.66	64.09 ± 11.74	62.86 ± 9.50	0.688
Gender				
Male	94 (69.6)	8 (8.5)	86 (91.5)	0.454
Female	41 (30.4)	3 (7.3)	38 (92.7)	
Differentiation				
Well	23 (17)	3 (13)	20 (87)	0.515
Moderately	54 (40)	5 (9.3)	49 (90.7)	
Poorly	55 (40.7)	3 (5.5)	52 (94.5)	
Missing	3 (2.2)	—	3 (100)	
Lymphovascular invasion				
Yes	106 (78.5)	8 (7.5)	98 (92.5)	0.548
No	27 (20)	3 (11.1)	24 (88.9)	
Missing	2 (1.5)	—	2 (100)	
pN stage				
0	47 (34.8)	5 (10.6)	42 (89.4)	
1	34 (25.2)	0	34 (100)	
2	20 (14.8)	4 (20)	16 (80)	0.068
3	31 (23)	2 (6.5)	29 (93.5)	
Missing	3 (2.2)	—	3 (100)	
pT stage				
1	8 (5.9)	0 (0)	8 (100)	
2	17 (11.1)	2 (11.8)	15 (88.2)	0.744
3	74 (54.8)	7 (9.5)	67 (90.5)	
4	31 (22.9)	2 (6.5)	29 (93.5)	
Missing	5 (3.7)	—	5 (100)	
Tumor location				
Distal esophagus	12 (9)	0	12 (100)	0.636
Proximal stomach	69 (51)	5 (7.2)	64 (92.8)	
Middle stomach	27 (20)	3 (11.1)	24 (88.9)	
Distal stomach	23 (17)	3 (13)	20 (87)	
Whole stomach (Linitis plastica)	4 (3)	0	4 (100)	
Surgical margin				
Positive	26 (19.3)	3 (11.5)	23 (88.5)	0.500
Negative	107 (79.2)	8 (7.5)	99 (92.5)	
Missing	2 (1.5)	—	2 (100)	
Clinical stage				
IB	7 (5.2)	0	7 (100)	0.672
IIA	8 (5.9)	1 (12.5)	7 (87.5)	
IIB	9 (6.7)	0	9 (100)	
IIIA	22 (16.3)	2 (9.1)	20 (90.9)	
IIIB	15 (11.1)	0	15 (100)	
IIIC	4 (3)	0	4 (100)	
IV	2 (1.5)	0	2 (100)	
Missing	68 (50.3)	8 (11.8)	60 (88.2)	
Surgical stage				
IA	3 (2.2)	0	3 (100)	0.408
IB	11 (8.1)	0	11 (100)	
IIA	29 (21.5)	5 (17.2)	24 (82.8)	
IIB	28 (20.7)	1 (3.6)	27 (96.4)	
IIIA	20 (14.4)	2 (10)	18 (90)	
IIIB	15 (11.1)	0	15 (100)	
IIIC	23 (17)	3 (13)	20 (87)	
IV	1 (0.7)		1 (100)	
Missing	5 (3.7)	—	5 (100)	
Peri‐neural invasion				
Absent	46 (34.1)	5 (10.9)	41 (89.1)	0.429
Present	87 (64.4)	6 (6.9)	81 (93.1)	
Missing	2 (1.5)	—	2 (100)	
Tumor regression grade				
TRG1	4 (3)	0	4 (100)	0.872
TRG2	47 (34.8)	3 (6.4)	44 (93.6)	
TRG3	52 (38.5)	3 (5.8)	49 (94.2)	
Missing	32 (23.7)	5 (15.6)	27 (84.4)	
Type of neoadjuvant therapy				
None	18 (13.3)	5 (27.8)	13 (72.2)	
RT + CHT	42 (31.1)	2 (4.8)	40 (95.2)	**0.006**
Only CHT	70 (51.9)	4 (5.7)	66 (94.3)	
Missing	5 (3.7)	—	5 (100)	
Histology subtype				
Diffuse	19 (14.1)	0	19 (100)	0.339
Intestinal	106 (78.5)	11 (10.4)	95 (89.6)	
Missing	10 (7.4)	—	10 (100)	

*Note:* *P* values below 0.05 are in bold.

### 5.1 HER‐2 Status

All 135 specimens underwent HER‐2 amplification testing, revealing HER‐2 amplification in 11 patients (8.1%). The baseline characteristics presented in Table 1 were comparable between the two groups of HER‐2 positive and HER‐2 negative patients. The only significant difference observed was in the type of neoadjuvant protocol utilized. This variation may be attributed to differences in tumor locations, which necessitate distinct treatment approaches, ranging from tumors located in the distal esophagus to those in the distal stomach. In the proximal stomach, 5 cases (7.2%) were HER‐2 positive and 64 (92.8%) were HER‐2 negative. Similarly, in the middle stomach, 3 cases (11.1%) were HER‐2 positive and 24 cases (88.9%) were Her2‐negative, while in the distal stomach, 3 cases (13%) were HER‐2 positive and 20 cases (87%) were HER‐2 negative. Distal esophagus tumors and tumors involving the whole stomach were all HER‐2 negative (100%). The *p* value of 0.636 indicates no statistically significant association between HER‐2 status and tumor location (see Figure S1). Among the 105 patients who received neoadjuvant therapy (chemotherapy or chemoradiation), six (5.7%) were found to be HER‐2 positive. In contrast, among the 30 patients who had not received any preoperative treatment, five (27.8%) were HER‐2 positive. The relationship between treatment modality and HER‐2 status was nearly statistically significant based on the Chi‐square test (*p* = 0.053). When considering the surgical stage, the frequency of HER‐2 positivity was observed to be 0 (0%) in stage I, 6 (10.5%) in stage II, 5 (8.6%) in stage III, and 0 (0%) in stage IV.

The median follow‐up time for patients was 39 months, during which 68 patients passed away. Table 3 provides survival outcomes in the total cohort and subgroups based on HER‐2 status. Regardless of HER‐2 status, the median OS was 30 months (95% CI: 21.2–38.7). The median OS for HER‐2 positive versus HER‐2 negative patients was 21 months (95% CI: 1.4–40.6) versus 31 months (95% CI: 22.9–39), respectively (*p* = 0.18). Throughout the follow‐up period, 42 patients (31.1%) experienced distant or local recurrence. The median DFS, regardless of HER‐2 status, was 25 months. The median DFS was 15 months (95% CI: 1.4–28.5) for HER‐2 positive patients and 28 months (95% CI: 13.8–42.1) for HER‐2 negative patients, with a *p* value of 0.25 (see Figure S2).

### 5.2 MSI Status

MSI testing eligibility was assessed for all included specimens, with 43 excluded due to tissue exhaustion from prior HER‐2 testing (*n* = 32) or inadequate DNA quality (*n* = 11). The remaining 92 MSI‐eligible specimens were prioritized for analysis, with complete MSI results obtained for 66 cases (3 MSI‐H (4.5%); 63 non‐MSI‐H (95.5%)) (Figure 3). The mean age of these patients was 63.17 ± 8.83, with a range of 36–84 years. MSI testing revealed MSI‐H in three patients (4.5%), all of whom tested negative for HER‐2. Statistical analysis indicated no significant association between HER2 positivity and MSI (*p* = 0.454). When considering the surgical stage, the MSI‐H cases were 1 in stage IB, 1 in stage IIA, and 1 in stage IIIA. The patients' characteristics are detailed in Table 2. While there were no statistically significant differences in most tumor characteristics, the average age of patients with MSI‐High tumors was 60.33 ± 1.15 years, compared with 63.30 ± 9.023 years for MSI‐Negative cases, showing a considerable difference (*p* = 0.034). For lymphovascular invasion (LVI), 2% of cases were in the MSI‐High group and 98% in the MSI‐Negative group; this difference was marginally statistically significant (*p* = 0.063). Perineural invasion (PNI) was observed in all MSI‐Negative cases, with no cases in the MSI‐High group, indicating a significant difference (*p* = 0.045).

**TABLE 2:** Baseline pathologic characteristics of MSI‐checked patients.

Characteristics	Number (percentage)	MSI high	MSI negative	*p*
Age				
Year	63.17 ± 8.83	60.33 ± 1.15	63.30 ± 9.023	**0.034**
Gender				
Male	44 (66.7)	1 (2.3)	43 (97.7)	0.210
Female	22 (33.3)	2 (9.1)	20 (90.9)	
Differentiation				
Well diff	15 (22.7)	0	15 (100)	0.497
Moderately diff	25 (37.9)	2 (8)	23 (92)	
Poorly diff	25 (37.9)	1 (4)	24 (96)	
Missing	1 (1.5)	—	1 (100)	
HER‐2 expression				
Positive	10 (15.1)	0	10 (100)	0.454
Negative	56 (84.9)	3 (5.4)	53 (94.6)	
Histological subtypes				
Intestinal	54 (81.8)	2 (3.7)	52 (96.3)	0.605
Diffuse	7 (10.6)	0	7 (100)	
Missing	5 (7.6)	—	5 (100)	
Clinical stages				
IB	5 (7.6)	1 (20)	4 (80)	0.846
IIA	2 (3)	0	2 (100)	
IIB	2 (3)	0	2 (100)	
IIIA	15 (22.7)	1 (6.7)	14 (93.3)	
IIIB	7 (10.6)	0	7 (100)	
IIIC	2 (3)	0	2 (100)	
IV	1 (1.5)	0	1 (100)	
Missing	32 (48.5)	1 (3.1)	31 (96.9)	
Surgical stage				
IA	1 (1.5)	0	1 (100)	0.737
IB	8 (12.1)	1 (12.5)	7 (87.5)	
IIA	17 (25.8)	1 (5.9)	16 (94.1)	
IIB	11 (16.7)	0	11 (100)	
IIIA	9 (13.6)	1 (11.1)	8 (88.9)	
IIIB	8 (12.1)	0	8 (100)	
IIIC	11 (16.7)	0	11 (100)	
Missing	1 (1.5)	—	1 (100)	
Tumor location				
Distal esophagus	7 (10.6)	1 (14.3)	6 (85.7)	0.351
Proximal stomach	41 (62.1)	1 (2.4)	40 (97.6)	
Middle stomach	9 (13.6)	0	9 (100)	
Distal stomach	9 (13.6)	1 (11.1)	8 (88.9)	
pT				
pT1	4 (6.1)	0	4 (100)	0.622
pT2	9 (13.6)	1 (11.1)	8 (88.9)	
pT3	38 (57.6)	2 (5.3)	36 (94.7)	
pT4	14 (21.2)	0	14 (100)	
Missing	1 (1.5)	—	1 (100)	
pN				
N0	26 (39.4)	2 (7.7)	24 (92.3)	0.578
N1	14 (21.2)	1 (7.1)	13 (92.9)	
N2	12 (18.2)	0	12 (100)	
N3	13 (19.7)	0	13 (100)	
Missing	1 (1.5)	—	1 (100)	
LVI				
Positive	51 (77.3)	1 (2)	50 (98)	0.063
Negative	15 (22.7)	2 (13.3)	13 (86.7)	
PNI				
Positive	37 (56.1)	0	37 (100)	**0.045**
Negative	29 (43.9)	3 (10.3)	26 (89.7)	
Surgical margins				
Positive	12 (18.2)	0	12 (100)	1
Negative	54 (81.8)	3 (5.6)	51 (94.4)	
Tumor regression grade				
TRG1	3 (4.5)	0	3 (100)	0.921
TRG2	21 (31.8)	1 (4.8)	20 (95.2)	
TRG3	27 (41)	1 (3.7)	26 (96.3)	
Missing	15 (22.7)	1 (6.6)	14 (93.4)	
Type of neoadjuvant therapy				
None	7 (10.6)	0	7 (100)	0.717
RT ± ChT	30 (45.4)	2 (6.7)	28 (93.3)	
Only ChT	27 (41)	1 (3.7)	26 (96.3)	
Missing	2 (3)	—	2 (100)	
Local recurrence				
Yes	1 (1.5)	0	1 (100)	0.826
No	65 (98.5)	3 (4.6)	62 (95.4)	
Distant recurrence				
Yes	19 (28.8)	0	19 (100)	0.260
No	47 (71.2)	3 (6.4)	44 (93.6)	

*Note:* *P* values below 0.05 are in bold.

In the distal esophagus, 1 case (14.3%) was MSI‐H, while 6 cases (85.7%) were MSI‐negative. In the proximal stomach, 1 case (2.4%) was MSI‐H compared with 40 cases (97.6%) that were MSI‐negative. No MSI‐H cases were observed in the middle stomach, where all 9 cases (100%) were MSI‐negative. In the distal stomach, 1 case (11.1%) was MSI‐H, and 8 cases (88.9%) were MSI‐negative. However, no statistically significant association between MSI status and tumor location was observed (*p* = 0.351) (see Figure S3).

During the study and patient follow‐up, no local or distant primary tumor recurrence was observed in the three patients with MSI‐H status. Pathologic treatment response data was available for 51 cases, revealing a poor pathologic response to pre‐surgical treatment in two patients with MSI‐H status: one with tumor regression grade 2 and the other with grade 3. The third patient had not received preoperative therapy.

**FIGURE 4:**
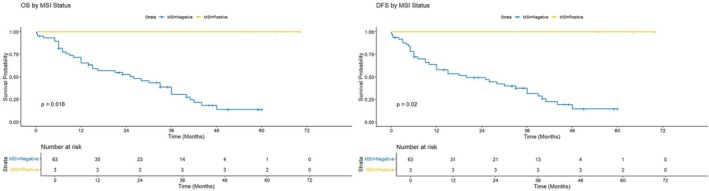
Overall and disease‐free survival based on MSI status. Hazard ratio (HR) could not be calculated since no events were observed in the MSI‐positive subgroup.

In the MSI cohort, the median follow‐up time of the survivors was 46 months, calculated using the reverse Kaplan–Meier method. As shown in Table 3, MSI‐negative patients had a median OS of 26 months, while no events occurred in the MSI‐positive group, indicating a significant difference (*p* = 0.018). Similarly, MSI‐negative patients had a median DFS of 20 months, while no events were reported in the MSI‐positive group, showing a significant difference (*p* = 0.020) (Figure 4).

**TABLE 3:** Survival outcomes in the total cohort and subgroups based on HER‐2 and MSI status.

Outcome	Overall (*n* = 135)	HER‐2 (*n* = 135)			MSI (*n* = 66)		
		Positive (*n* = 11)	Negative (*n* = 124)	*p*	Positive (*n* = 3)	Negative (*n* = 63)	*p*
Median overall survival (months)	30	21	31	0.18	No events	26	**0.018**
Median disease‐free survival (months)	25	15	28	0.24	No events	20	**0.020**

*Note:* *P* values below 0.05 are in bold.

## 6 Discussion

We conducted a study to investigate the clinicopathological characteristics and relevant survival outcomes of patients with gastric and GEJ adenocarcinoma due to the high incidence of gastric cancer in Iran when compared with other countries. Our research was focused on investigating the possibility of HER2‐Neu and MSI being potential targets for the treatment of gastric and GEJ adenocarcinoma.

Our findings are compatible with prior studies conducted in Iran [35, 36], which have also reported that the incidence of the disease is more common in males than females, with male‐to‐female ratios of 2:1, 2.3:1, and 3.75:1, respectively. These findings have also been reported in other studies, including a meta‐analysis [35, 36, 37, 38, 39, 40].

The most commonly affected sites in this study, as well as in other studies from Iran and Asian countries like Japan and South Korea, are gastric cardia and GEJ. Studies from the United States, India and Iran by Devesa et al. [3], Harikumar et al. [41], and Abdi‐Rad et al. [39] have observed a shift in the prevalence of gastric cancer towards the cardia/proximal stomach. This trend was also noted in the present study and may be associated with changes in *Helicobacter pylori* colonization, atrophic gastritis, dietary habits, lifestyle, obesity, and GE reflux [42].

The rate of Her2‐Neu positivity in gastric/GEJ cancers varies between continents. According to a meta‐analysis by Lei et al., the rate of positivity is slightly higher in Asian countries (19.52%) compared with European countries (16.91%) [38]. This variation in expression is also evident within Asian countries themselves, including Iran [35, 36, 43]. Shan et al. revealed a frequency of 9.8% in china [44], while Matsusaka et al.'s Japanese multicenter observational study demonstrated a 15.6% frequency of high Her2‐Neu expression [45]. In Iranian studies, geographical differences were observed, with Feizy et al. from Mazandaran reporting a 24% frequency [43], while studies from Tehran showed different results: Basi et al. reported an 11.3% frequency in their cross‐sectional study [46], Moradi et al. reported a 28% frequency [35], and Rakhshani et al. noted a 13% HER‐2 positivity rate [36]. Finally, the frequency observed in the present study conducted at a tertiary care institute in Tehran was 8.1%.

In our study HER‐2 positivity rate was 8.1%. HER‐2 status showed no significant association with other tumor characteristics or MSI status. Patients with HER‐2 overexpression showed a lower mean OS and DFS when compared with those who had negative HER‐2 tumors. This was observed even after neoadjuvant therapy and adjuvant chemotherapy, which was administered to over 70% of the patients. Based on a systematic review of 49 studies involving 11,337 patients, individuals who had positive HER‐2 expression had a 3‐year DFS rate of 58%. This is in contrast to those with negative HER‐2 expression, who had a rate of 86%. The survival rate without disease for 3 years varied between 50%–88% and 62%–97%, respectively. The median and the 5‐year OS was 21 versus 33 months and 42% versus 52% in the HER2‐positive and HER2‐negative groups, respectively. These results suggest that tumors that are HER‐2 positive are linked to advanced disease and lower rates of survival, even with neoadjuvant and adjuvant chemotherapy. Therefore, it is necessary to implement targeted interventions, which may bring some hope [47].

Research has shown that MSI plays a significant role in gastric cancer. Resected GE cancers have an incidence of MSI‐H that ranges between 6% and 24%, and the incidence of MSI‐H in the MAGIC trial was 6.6% [48]. Cristescu et al. found that tumors with MSI were hypermutated tumors of the intestinal subtype in the antrum. This resulted in a more favorable overall prognosis when compared with tumors of the mesenchymal type [49]. Similarly, Liu et al. discovered that 58.3% of their gastric cancer cohort tested positive for MSI, suggesting that MSI accumulation in intestinal dysplasia and gastric mucosal metaplasia could be an early molecular event in gastric carcinogenesis [50]. In our study of 66 patients, we found that the MSI‐H rate was 4.5%, and all three MSI‐high tumors were located in the stomach and cardia. One patient did not receive any neoadjuvant therapies, and the other two patients with MSI‐H did not respond well to neoadjuvant treatments. However, the long‐term outcomes showed that MSI‐H cases experienced no recurrences during the follow‐up time.

This aligns with the findings of Cai et al., who concluded that high MSI‐H is associated with poorer histological regression following neoadjuvant chemotherapy in patients with clinical stage III gastric cancer. However, despite this attenuated treatment response, patients with MSI‐H had better survival rates compared with those with MSI‐L tumors. Additionally, TRG evaluation showed prognostic significance in patients with MSI‐L, but not in those with MSI‐H [51]. Additionally, Vos et al. showed patients with MSI‐H tumors had better survival compared with MSS tumors whether given chemotherapy or treated with surgery alone [52]. Moreover, Kohlruss et al. [53] conducted a study on 760 gastric cancer patients who were undergoing preoperative platinum/5‐fluorouracil‐based chemotherapy. The aim was to investigate the prognostic significance of Epstein–Barr virus (EBV) infection, MSI‐H, and MSI‐L. The study revealed that patients with EBV‐positive tumors had the best OS, followed by those with MSI‐H tumors. On the other hand, MSI‐L tumors were significantly associated with worse OS.

Contrastingly, an exploratory analysis of the MAGIC trial showed that resectable GE cancers with MSI‐L or MSS tumors tend to have better survival rates compared with those with MSI‐H tumors when undergoing perioperative chemotherapy combined with surgery. However, adding preoperative chemotherapy to surgery provided no benefits for MSI‐H cases, and treatment‐related deaths from this chemotherapy were the main reason for the lower survival rates observed in those receiving the treatment [30]. Similarly, in a study by Hashimoto et al., MLH1 and PD‐L1 expression were examined in 285 gastric cancer patients treated with or without preoperative chemotherapy. MLH1‐negative tumors (9.8%) were predominantly MSI‐H (85.7%) and frequently exhibited high PD‐L1 expression. MLH1‐negative patients showed poorer chemotherapy response and had significantly longer RFS without chemotherapy compared with MLH1‐positive patients, concluding that MLH1 loss is linked to chemoresistance and suggested immune checkpoint inhibitors as a preferable treatment for MLH1‐negative gastric cancer [54]. The discrepancy between studies on the response and survival of MSS/MSI‐L and MSI‐H cases may be attributed to three key hypotheses. First, variations in tumor stage across studies likely influence outcomes, with less advanced tumors being overrepresented in some studies (e.g., MAGIC trial), potentially leading to divergent results. Second, differences in NAC regimens, such as fluorouracil/platinum versus taxane‐based treatments, may account for MSI‐H chemoresistance patterns, with taxane‐based regimens potentially offering survival benefits for MSI‐H patients. Finally, variability in diagnostic criteria for MSI status, including differences in PCR and IHC (e.g., Hashimoto et al.) detection methods and panels, reduces population comparability and may contribute to conflicting findings.

Our study aligns with previous reports in terms of the prognostic information that can be inferred from MSI status. However, some differences in frequency suggest that our cohort had lower rates of MSI positivity. Despite the lower frequency of MSI‐H status, it is worth noting that neoadjuvant therapy has been shown to have little impact on the response of MSI‐H patients, as seen in the MAGIC trial [30]. Therefore, the lower frequency of MSI positivity in our study cannot be attributed to the influence of neoadjuvant therapy on MSI status. This is consistent with findings on the effects of treatment on MSI status in colorectal cancer [55, 56]. Moreover, technical issues and differences in assessment methods may have contributed to the variation observed. There are two standard reference methods recommended for detecting MMR deficiency in tumors, which include PCR to detect affected genes and loss of MMR protein expression by IHC [57]. The MSI phenotype can be determined using PCR methods and criteria such as Pentaplex and HT17‐Repeat, with the 17‐mononucleotide repeat of HSP110 (HT17) being crucial for improving current standard molecular methods for detecting MSI in colorectal cancer [58]. In our study, we utilized pentaplex PCR to detect MMR protein expression. The reason for this is that gastric cancer is a heterogeneous disease in terms of its genome, and the underlying mutational landscape of GEJ and gastric cancers can vary among different populations. This issue can limit the external validity of using findings from other studies in our population and justifies the need for population‐specific assessments.

The strengths of this study include its comprehensive approach, which encompasses a large cohort of patients with gastric or GEJ adenocarcinoma over a nearly five‐year period, rigorous histopathological and molecular analyses using HER‐2 and MSI markers, and a detailed examination of clinicopathological characteristics and survival outcomes.

Our study has several limitations. First, it was a retrospective single‐center study. Second, the considerable time elapsed before sample examination may have impacted the positivity rates of HER‐2 and MSI, highlighting the need for analysis of more recent samples. Third, budget constraints restricted MSI testing to approximately two‐thirds of the eligible cases. Additionally, incomplete patient records and a lower HER‐2 positivity rate (less than 10%) in adenocarcinoma compared with other studies posed challenges. This lower rate may be due to the fact that most patients receive neoadjuvant treatment, which could affect HER‐2 expression. Furthermore, the absence of pre‐treatment pathology samples prevented a detailed analysis of HER‐2.

## 7 Future Directions

Recent study findings highlight the promise of ICI‐based neoadjuvant therapy in gastric or GEJ adenocarcinoma and encourage further large‐scale randomized trials. A meta‐analysis of 21 prospective phase I/II studies involving 687 patients evaluated the efficacy and safety of ICI‐based neoadjuvant therapy in locally advanced gastric cancer. The pathological complete response (pCR) rate was 21%; the major pathological response (MPR) rate was 41%; and the R0 resection rate was 94%, with the highest efficacy observed in ICI plus chemoradiotherapy regimens. Patients with MSI‐H or high PD‐L1 expression benefited the most. Grade 3 or higher toxicity was lower than that in traditional neoadjuvant chemotherapy trials [59].

The phase 3 CheckMate 649 trial evaluated nivolumab plus chemotherapy versus chemotherapy alone as first‐line treatment for advanced HER2‐negative gastric/GEJ/esophageal adenocarcinoma. Among 1581 randomized patients, nivolumab plus chemotherapy significantly improved OS and progression‐free survival (PFS) in PD‐L1 CPS ≥ 5 patients, with additional OS and PFS benefits in CPS ≥ 1 and all patients. After 3 years of follow‐up, nivolumab plus chemotherapy continued to demonstrate clinically meaningful OS improvement [60].

The treatment landscape of HER‐2 positive gastric cancer is undergoing a transformative phase, driven by the introduction of novel anti‐HER2 agents, combination therapies, and immunotherapy that show strong preclinical and clinical promise [61].

An ongoing Phase 2 clinical trial (NCT05034887) is exploring Trastuzumab Deruxtecan (T‐DXd) in neoadjuvant and adjuvant treatment for HER‐2 positive gastric and GEJ adenocarcinoma. The study evaluates T‐DXd monotherapy (6.4 mg/kg every 21 days for three cycles) and a combination regimen with capecitabine and durvalumab, given both pre‐ and postoperatively. Subgroups include patients with HER‐2 overexpression and low HER‐2 expression. Results from this trial may guide more effective, personalized treatment strategies for HER‐2 positive gastric cancers.

Another ongoing study (NCT02205047) is a randomized phase II trial investigating trastuzumab alone or in combination with pertuzumab as neoadjuvant therapy for HER‐2 positive gastric and GEJ adenocarcinoma. Patients are screened centrally for HER‐2 positive status and randomized in a 1:2:2 ratio between a control arm, trastuzumab monotherapy, and trastuzumab plus pertuzumab. This study leverages the concept of dual HER‐2 blockade, where trastuzumab and pertuzumab target different domains aiming to refine neoadjuvant strategies and improve outcomes for HER‐2 positive gastric cancer.

These studies highlight the potential of combining ICIs, anti‐HER2 agents, and novel regimens to advance treatment strategies and improve survival in gastric or GEJ adenocarcinoma.

## 8 Conclusion

This study provides a comprehensive analysis of gastric and GE adenocarcinoma cases, highlighting important clinical and pathological features. Although HER‐2 overexpression was identified in 8.1% of patients, which is lower than that in Western and North American studies but more similar to East Asia, its linkage to unfavorable outcomes is preserved in our patients with gastric and GE adenocarcinoma. The frequency of MSI‐H is lower than other reports worldwide, but its prognostic information is in line with previous reports. HER‐2 positivity showed no significant association with tumor characteristics or MSI status. However, a trend toward improved OS and DFS was observed in HER‐2 negative cases; although this was not significant. MSI‐H was detected in 4.5% of cases and was significantly associated with reduced perineural invasion and markedly improved OS and DFS, with no recurrences observed during follow‐up. Since MSI‐H status was highly infrequent in our population, immunotherapy may not be a beneficial treatment for a significant fraction of Iranian patients with gastric adenocarcinoma. However, a minority may still benefit from it. These findings underscore the distinct prognostic implications of HER‐2 and MSI status, highlighting the need for molecular profiling to guide personalized treatment strategies for gastric and GE adenocarcinomas.

## Author Contributions

Conceptualization: Samaneh Salarvand, Reza Ghalehtaki. Methodology: Samaneh Salarvand, Abbas Mohammadi, Reza Ghalehtaki. Software: Somayeh Jolany‐Vangah. Data curation: Samaneh Salarvand, Reza Shahsiah, Farzaneh Bagheri, Mahsa Gholizadeh, Vahid Soleymani, Amirmohsen Jalaeefar, Romina Abyaneh, Reza Ghalehtaki. Investigation: Samaneh Salarvand, Reza Shahsiah, Farzaneh Bagheri, Vahid Soleymani, Amirmohsen Jalaeefar, Mohammad Shirkhoda, Reza Ghalehtaki. Validation: Samaneh Salarvand, Reza Shahsiah, Amirmohsen Jalaeefar, Reza Ghalehtaki. Formal analysis: Reza Ghalehtaki, Romina Abyaneh. Supervision: Samaneh Salarvand, Ebrahim Esmati, Marzieh Lashkari, Amirmohsen Jalaeefar. Visualization: Somayeh Jolany‐Vangah, Mohammad Shirkhoda. Project administration: Samaneh Salarvand, Ebrahim Esmati, Marzieh Lashkari, Reza Ghalehtaki. Writing – original draft: Samaneh Salarvand, Abbas Mohammadi, Farzaneh Bagheri, Seraj Zareh‐Dehabadi, Vahid Mehrtash, Moones Toosang, Reza Ghalehtaki. Writing – review and editing: Samaneh Salarvand, Abbas Mohammadi, Ebrahim Esmati, Marzieh Lashkari, Seraj Zareh‐Dehabadi, Vahid Mehrtash, Moones Toosang, Romina Abyaneh, Reza Ghalehtaki.

## Ethics Statement

The local ethics committee reviewed and approved this study (Ethics code: IR.TUMS.VCR.REC.1397.020, IR.TUMS.IKHC.REC.1397.325). The patient gave written informed consent to the publication of case details and the use of images.

## Conflicts of Interest

The authors declare no conflicts of interest.

## Supporting information


**Data S1:** Supporting Information.

## Data Availability

The data that support the findings of this study are available from the corresponding author upon reasonable request.
