# Supplementary material for: Human Epidermal Growth Factor Receptor 2 [HER‐2/neu] Amplification and Microsatellite Stable Status in Gastric and Gastroesophageal Adenocarcinoma: Assessing Frequency and Prognostic Implications at the Cancer Institute of Iran
Source: Cancer Rep (Hoboken). 2025 Aug 25;8(8):e70314. doi: 10.1002/cnr2.70314 (PMC12375872; doi:10.1002/cnr2.70314)
Supplement: Supplementary file 1 — Data S1: Supporting Information. [file CNR2-8-e70314-s001.docx]

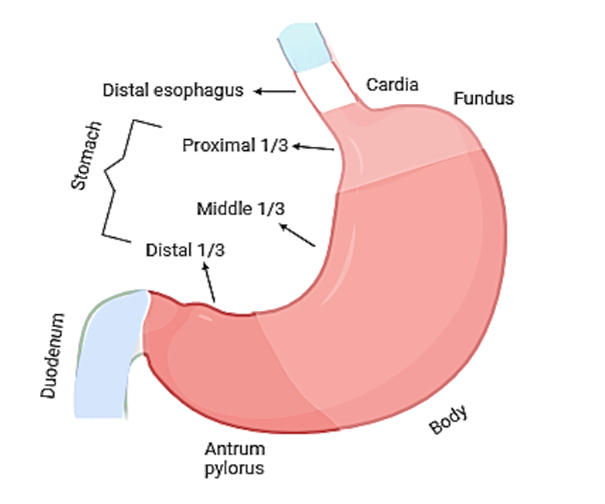


Figure S1 The Figure represents HER-2 positivity based on tumor location (Designed and prepared by the authors using Biorender). A color gradient has been used for interpretation, where darker shades represent higher incidences, white indicates no HER-2 positivity, and light blue denotes regions not assessed.


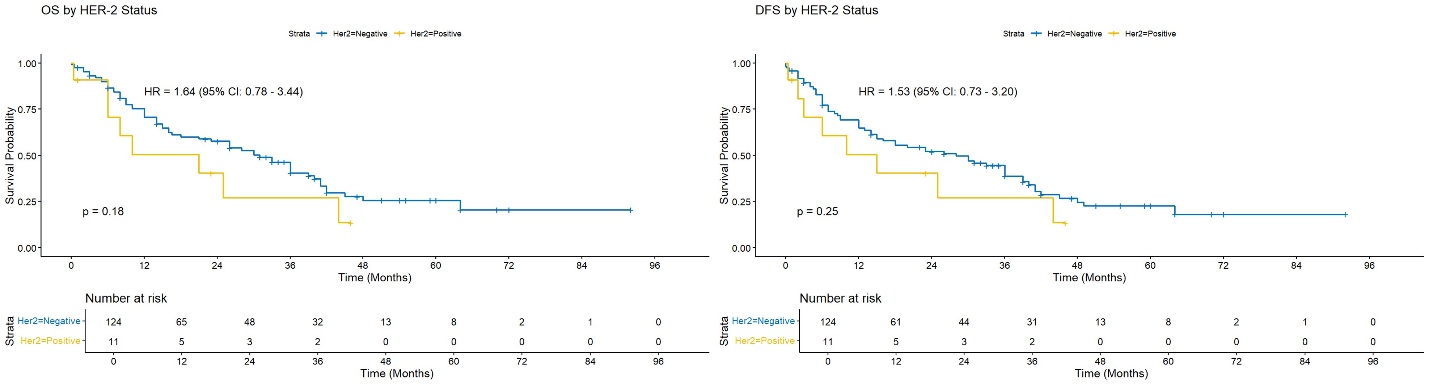


Figure S2 Overall survival (OS) and disease-free survival (DFS) based on HER-2 status


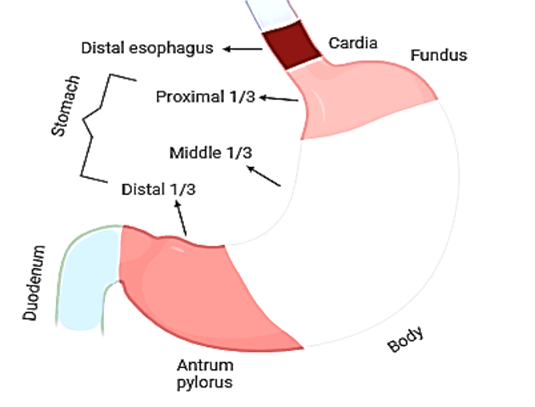


Figure S3 The figure represents MSI-H rate based on primary tumor location (Designed and prepared by the authors using Biorender). A color gradient has been used for interpretation, where darker shades represent higher rates, white indicates no MSI-H, and light blue denotes regions not assessed.
